# Scaling up of safety and quality improvement interventions in perioperative care: a systematic scoping review of implementation strategies and effectiveness

**DOI:** 10.1136/bmjgh-2022-010649

**Published:** 2022-10-26

**Authors:** Michelle C White, Shalini Ahuja, Kimberly Peven, Susanna Ritchie McLean, Dina Hadi, Ijeoma Okonkwo, Olivia Clancy, Maryann Turner, Jaymie Claire Ang Henry, Nick Sevdalis

**Affiliations:** 1 Department of Anesthesia, Great Ormond Street Hospital, London, UK; 2 Centre for Global Health, King’s College London, London, UK; 3 University College London, London, UK; 4 Methodologies Research Division, Faculty of Nursing Midwifery and Pallative Care, London, UK; 5 Florence Nightingale Faculty of Nursing and Midwifery, King’s College London, London, UK; 6 Department of Anesthesia, Birmingham Women’s and Children’s NHS Foundation Trust, Birmingham, UK; 7 Department of Anesthesia, Whittington Hospital, London, UK; 8 Department of Anaesthesia, Alder Hey Children’s Hospital, London, UK; 9 Department of Anaesthesia, Manchester Children’s Hospital, Manchester, UK; 10 Department of Anaesthesia, Children's Hospital at Westmead, Westmead, New South Wales, Australia; 11 Department of Surgery, Florida Atlantic University, Boca Raton, Florida, USA; 12 Centre for Implementation Science, Health Service and Population Research Department, Institute of Psychiatry, Psychology and Neuroscience, King's College London, London, UK

**Keywords:** Surgery, Health services research, Health systems, Review

## Abstract

**Background:**

Globally, 5 billion people lack access to safe surgical care with more deaths due to lack of quality care rather than lack of access. While many proven quality improvement (QI) interventions exist in high-income countries, implementing them in low/middle-income countries (LMICs) faces further challenges. Currently, theory-driven, systematically articulated knowledge of the factors that support successful scale-up of QI in perioperative care in these settings is lacking. We aimed to identify all perioperative safety and QI interventions applied at scale in LMICs and evaluate their implementation mechanisms using implementation theory.

**Methods:**

Systematic scoping review of perioperative QI interventions in LMICs from 1960 to 2020. Studies were identified through Medline, EMBASE and Google Scholar. Data were extracted in two phases: (1) abstract review to identify the range of QI interventions; (2) studies describing scale-up (three or more sites), had full texts retrieved and analysed for; implementation strategies and scale-up frameworks used; and implementation outcomes reported.

**Results:**

We screened 45 128 articles, identifying 137 studies describing perioperative QI interventions across 47 countries. Only 31 of 137 (23%) articles reported scale-up with the most common intervention being the WHO Surgical Safety Checklist. The most common implementation strategies were training and educating stakeholders, developing stakeholder relationships, and using evaluative and iterative strategies. Reporting of implementation mechanisms was generally poor; and although the components of scale-up frameworks were reported, relevant frameworks were rarely referenced.

**Conclusion:**

Many studies report implementation of QI interventions, but few report successful scale-up from single to multiple-site implementation. Greater use of implementation science methodology may help determine what works, where and why, thereby aiding more widespread scale-up and dissemination of perioperative QI interventions.

**WHAT IS ALREADY KNOWN ON THIS TOPIC**

- At the point of care, various quality improvement (QI) interventions are delivered to reduce surgical morbidity and mortality.
- These QI interventions are critical since, worldwide, poor-quality services cause more deaths than a lack of access to healthcare.
- However, there is still poor knowledge about how to scale up QI interventions. Because resources are limited, surgical quality and safety are generally poor, and surgical outcomes are much worse in some of the low/middle-income countries (LMICs), the need to reduce the implementation gap in scaling is even higher than in high-income countries.

**WHAT THIS STUDY ADDS**

- To the best of our knowledge this is one of the first studies to assess the scale-up of perioperative point-of-care patient safety interventions in LMICs using established implementation frameworks.
- Interventions such as enhanced recovery after surgery and antimicrobial stewardship (including reducing surgical site infections) are currently under-represented in LMICs; our study reports a possibility of using theoretical implementation methodologies to better understand the relationship between contextual factors and success, as well as to discover scale-up facilitators and impediments.

**HOW THIS STUDY MIGHT AFFECT RESEARCH, PRACTICE OR POLICY**

- In LMICs, surgical safety science is shifting its focus from an evidence gap to a scalability gap, which will have an impact on effective coverage of health services.

## Introduction

Since the publication of the Institute of Medicine’s Report ‘To Err is Human’,^1^ much progress has been made in improving surgical outcomes by reducing errors and improving the quality and safety of patient care. Many quality improvement (QI) interventions now exist that are proven to reduce surgical morbidity and mortality. Such QI interventions are important because globally, more deaths are due to lack of quality rather than lack of access to healthcare.^2^ In various healthcare and country contexts, the discussion about the quality of health services versus lack of access to healthcare extends beyond surgery. However, knowledge of how to successfully implement QI interventions at scale remains lacking.^3 4^ Over a decade ago, this failure to rapidly deploy QI technologies and practices widely to improve population health was described as a ‘form of waste that donors, researchers, clinicians, and most of all, communities in developing nations cannot afford.’^5^ Recently, the challenges of scale-up were highlighted by the Enhancing PeriOperative Care after High risk surgery trial. Despite strong evidence in favour of the intervention at local level, the benefits could not be replicated when scaled up at the national level in the UK.^6 7^ According to the WHO, the failure to expand successful pilot or demonstration projects around the world is a ‘major failure in global health’.^8^ There is increasing recognition that guideline publications, policy reform and training alone are insufficient to achieve successful and sustainable scale-up.^9–11^ For complex interventions, understanding the factors that facilitate or hinder implementation is critically important. The focus of surgical safety science is thus shifting from addressing an evidence gap to an implementation gap in scalability.^12^

In low/middle-income countries (LMICs), the imperative to close the implementation gap in scalability is even greater than in high-income countries (HICs) because resources are limited, surgical quality and safety are often poor, and outcomes are significantly worse.^13–15^ Failure to close the implementation gap means that potential ‘solutions’ for improving the quality of care risk remaining hidden in pockets around the globe, flourishing locally without reliably reaching those in need elsewhere.^5^ In 2019, the WHO Global Ministerial Patient Safety Summit declared that healthcare systems globally must urgently focus on the principles of implementation science if the momentum of the global patient safety movement is to be realised.^16^ This notion was also highlighted by the 2021 GlobalSurg-3 Study which shows that while surgical complication rates after cancer surgery are similar globally, the 30-day mortality is significantly greater in LMICs.^17^ The authors estimated that improving infrastructure and perioperative care would save an additional 10 lives per 100 complications. Since many proven perioperative QI interventions exist, one of the greatest challenges currently facing the global surgical community is how to implement them successfully at scale in LMICs.

Implementation science is a field of applied health research that could help address this challenge. Implementation science is a form of health policy and systems research that is used to study and support the scale-up and integration of interventions into health systems at the regional and national level (online supplemental appendix 1).^18^ Broadly, efforts have been made to define, classify and systematise the implementation science literature with the aim of enabling researchers and clinicians to better understand the current evidence base, identify knowledge gaps, and more clearly report implementation strategies and outcomes. Powell *et al*^19^ have developed a taxonomy (Expert Recommendations for Implementing Change: ERIC) to define implementation strategies, and to move away from confusing or unclear language when reporting results. Likewise, a framework for conceptualising and evaluating implementation outcomes has been proposed,^20^ which is used by the WHO as a means of defining ‘successful implementation’ (online supplemental appendix 4).

10.1136/bmjgh-2022-010649.supp1 Supplementary data

This systematic scoping review aims to address the current gap in understanding whether and how interventions proven clinically effective in reducing mortality and morbidity postoperatively have been scaled up in surgical settings in LMICs. We used concepts from the science of implementation, first to identify all perioperative QI interventions applied at scale in LMICs; and second, to evaluate the mechanisms and effectiveness of their scaled implementation using theoretical frameworks.

## Methods

We conducted a systematic scoping review of perioperative QI interventions in LMICs. The aim of a systematic scoping review is to define and map the current evidence base for an emerging field. Such reviews can address broad questions and include varied methodologies; evidence is captured through systematic and comprehensive search, analysis and reporting.^21^ We followed the recommended five-step approach for such reviews, as detailed below.^22 23^ The review protocol was registered a priori with the Open Science Framework (https://osf.io/s6y98).

### Review question

The research question was developed by using the SPIDER question format tool^24^: Sample, Phenomenon of Interest, Design, Evaluation and Research type. The SPIDER tool was chosen since it was designed mainly for qualitative/mixed-methods studies and therefore aligns best with the studies of interest in LMICs. The study samples belong to populations (including children and adults) from LMICs. LMICs were defined using World Bank definitions, which are based on gross national income (https://datahelpdesk.worldbank.org/knowledgebase/articles/906519-world-bank-country-and-lending-groups).

The phenomenon of interest was defined as perioperative QI interventions, which are delivered at the point of care (ie, at the clinical–patient interface level). The search, therefore, excluded interfaces between health systems, such as implementing a database, designing and implementing electronic medical records, or simply engaging in a training programme. The list of perioperative QI interventions was derived from the UK Royal College of Anesthetists ‘audit recipe book’ for perioperative safety and quality improvement.^25^ While the ‘audit recipe book’ was predominantly designed for use in HICs, in the absence of a list designed for LMICs, it nonetheless represents a clinically relevant, established and comprehensive list on which to base the search strategy.

### Search strategy

Two electronic databases—Medline and EMBASE—were searched from January 1960 to April 2020. Searches produced through Google Scholar, which is identified as a powerful search mechanism,^26^ were also included. The search strategy (online supplemental appendix 3) consisted of angling terms related to “surgery”, “point of care interventions”, “LMICs”, “scale-up” and a list of perioperative QI interventions which included terms such as: checklist, triage, early warning score, protocol, guideline, quality improvement, pathway, bundle, fasting, thromboprophylaxis, patient admission, airway and failure to rescue. Backward and forward citation searches of identified papers were also carried out.

### Study selection

A two-stage approach was taken. Stage 1 selection was designed to identify *all* perioperative QI interventions implemented in LMICs, and stage 2 aimed to identify those implemented at scale. Since there is no universally agreed definition of ‘scale-up’ in the healthcare literature, we defined it using an iterative approach—as follows:

*Stage 1*: inclusion was based on study title, abstract and keywords. Screening was based on the phenomenon of interest, which was perioperative point-of-care QI interventions implemented in LMICs. Studies were included if they were original human studies that reported primary data. Reviews and secondary data reports were excluded. QI interventions beyond the point of care were excluded, such as implementing a database, electronic medical records or ad-hoc training programmes for healthcare workers. However, if ‘training’ was part of implementing the intervention (ie, training in how to use a checklist), then it was included. QI interventions implemented outside hospitals were excluded. Additionally, cross-sectional studies that only assessed knowledge, attitudes and behaviours of health workers for the QI interventions were also excluded. There were no restrictions imposed on types of evaluation, study designs or language.

Screening was done through the online software Rayyan. Three reviewers (MCW, SA, NS) screened a quarter of the search results in triplicate to calibrate our judgement of the inclusion criteria and establish reliable consensus for quality assurance purposes. Two authors (MCW and SA) then trained six further reviewers (IO, OC, SRM, DH, MT, JCAH) who screened the remaining articles, initially in duplicate with MCW to ensure calibration and provide quality assurance, while SA acted as arbitrator.

*Stage 2*: once we had identified all perioperative QI interventions reported in LMICs (stage 1), we identified the median number of hospital sites where QI interventions were applied (median=1; IQR 1–2) and pragmatically decided to define scale-up as per the upper quartile, that is, implementing an intervention in three or more sites. Stage 2 criteria were therefore not defined a priori in the registered protocol. The full texts of all articles identified by stage 2 criteria were retrieved.

### Data extraction and management

At stage 1, five reviewers (IO, OC, SRM, DH, MT) extracted descriptive data including country of origin, type of QI intervention and number of hospital sites where the intervention was applied.

At stage 2, two teams of reviewers (IO and OC; DH and SRM) extracted data on implementation strategies using the ERIC framework^19^; implementation success using the implementation outcomes framework^18 20^; and scale-up approaches using Yamey’s^27^ and Barker *et al*’s^11^ theoretical scale-up frameworks. Brief descriptions of these frameworks and rationale for their application were as follows:

- The *ERIC framework*^19^ consists of 73 discrete implementation strategies grouped into nine domains (online supplemental appendix 5): use evaluative and iterative strategies (n=10), provide interactive assistance (n=4), adapt and tailor to context (n=4), develop stakeholder inter-relationships (n=17), train and educate stakeholders (n=11), support clinicians (n=5), engage patients/service users (n=5), use financial strategies (n=9) and change infrastructure (n=8). This is currently the best-established framework for detailed analysis of strategies to support implementation, based on systematic review of the healthcare evidence base and Delphi consensus. We have recently applied this framework successfully to analyse implementation of surgical safety interventions in LMICs, including the WHO Surgical Safety Checklist^28^ and the use of audit and feedback to reduce surgical site infections (SSIs).^29^
- *Eight implementation outcomes* are defined in the implementation science evidence base^20^ and adopted by the WHO^18^: appropriateness, adoption, acceptability, feasibility, fidelity, implementation cost, penetration/reach and sustainability. This offers a currently agreed framework for defining the results of the process of implementing an intervention. We have recently shown that these outcomes are relevant to surgical safety interventions.^28 29^
- *Yamey’s framework*^27^ is designed for the scale-up of global health interventions. It consists of 13 discrete strategies grouped into six key components known to influence scale-up success: innovation characteristics, implementers, delivery strategy, adopting community, sociopolitical context and research context.
- *Barker et al’*s *framework*^11^ is based on lessons learnt from large-scale QI initiatives in Africa. It consists of 14 discrete strategies, grouped into three key components known to influence scale-up success: phase of setting scale-up, environmental factors/mechanisms to foster adoption and infrastructure to support scale-up.

Data were extracted in duplicate by OC and IO (implementation strategies and outcomes), and SRM and DH (scale-up strategies) with MCW acting as an arbitrator for both groups.

### Quality assessment of the included studies

Risk of bias was assessed by IO and OC using the QualSyst tool,^30^ which is designed to assess both quantitative and qualitative studies, including observational studies. Mixed-methods studies underwent both quantitative and qualitative assessments. Because a scoping search did not identify many randomised trials, we decided a priori that no study would be excluded based on study design or risk of bias.

## Results

**Figure 1:**
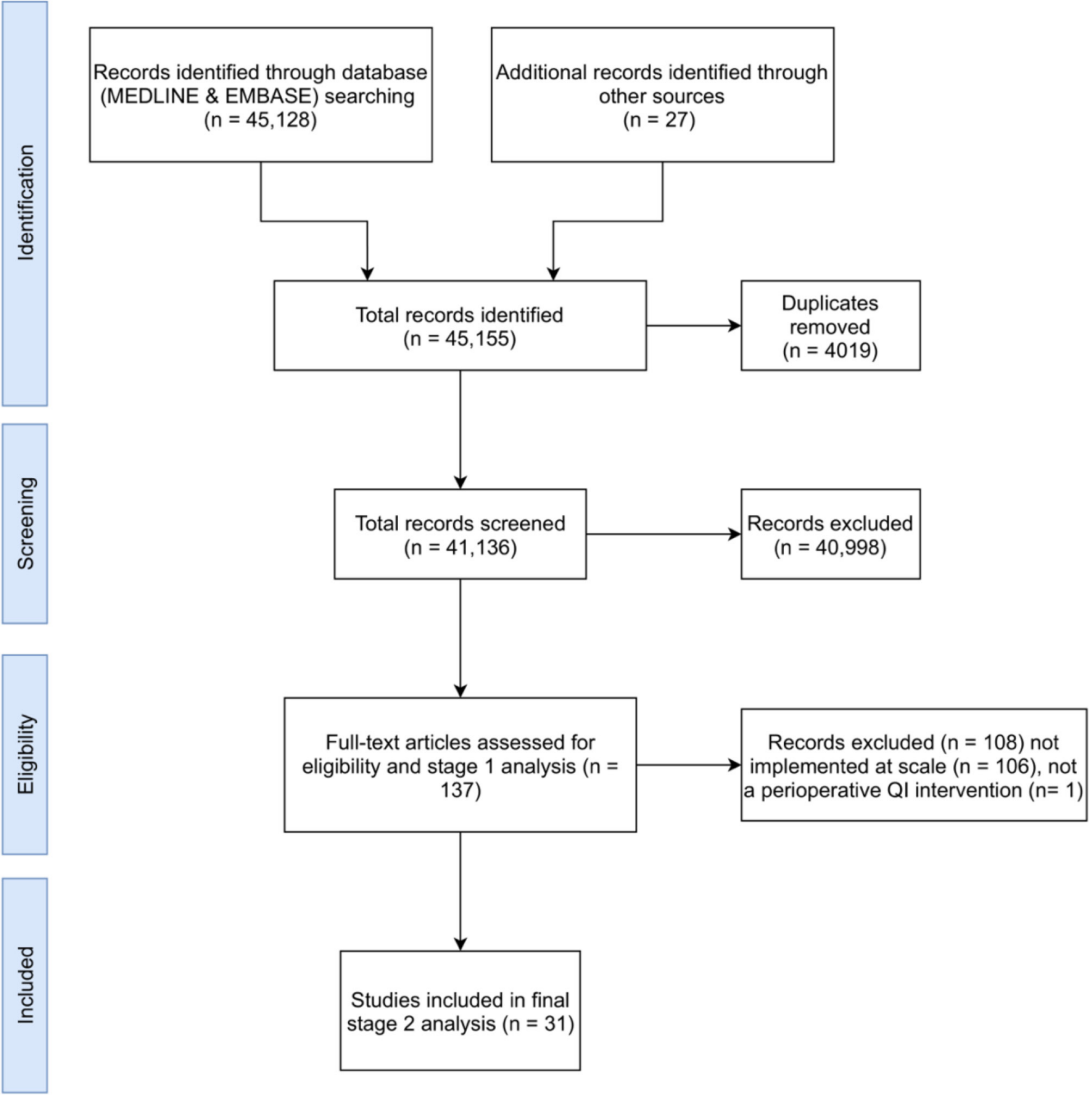
PRISMA diagram. PRISMA, Preferred Reporting Items for Systematic Reviews and Meta-Analyses; QI, quality improvement.

We screened 41 136 citations and included 137 papers reporting implementation of 144 point-of-care perioperative QI interventions at stage 1 of the review process (figure 1).

Stage 1 analysis revealed that the most common QI interventions implemented in LMICs were: the WHO Surgical Safety Checklist (including safe birth checklists) (44 of 144), prevention and management of SSIs and interventions aiming to improve antimicrobial stewardship (AMS) (33 of 144), and enhanced recovery after surgery (ERAS) (27 of 144) (table 1). Most of the interventions (106 of 137, 77%) were implemented in two or fewer sites.

**Table 1:** Type of QI intervention and frequency of implementation: overall and at scale (three sites or more)

Type of QI intervention	Scale-up sites (n=31)	Implementation sites (144 intervention types from 137 studies)
ERAS	1	27
Other	4	40
SSI/antimicrobial stewardship	5	33
Surgical checklist (including maternal/birth)	21	44

ERAS, enhanced recovery after surgery; QI, quality improvement; SSI, surgical site infection.

The median number of hospital sites undergoing QI implementation per study was 1 (range=1–120; IQR 1–2).

For stage 2 selection and final analysis, we defined scale-up as implementation at three or more sites based on the IQR of the number of study sites identified in stage 1. Therefore, studies implementing QI interventions at fewer than three sites did not meet the criteria for scale-up and were excluded. This resulted in 31 studies for final analysis (figure 1). Of these, 20 (65%) had a first author with an LMIC institutional affiliation and 11 (35%) with HIC institutional affiliation. Similarly, of last authors, 19 of 31 (61%) had LMIC institutional affiliations and 12 of 31 (39%) HIC affiliations.

Twenty-one out of 31 studies focused on safety checklists (including safe birthing checklists),^31^ ^32^ five on SSI/AMS,^33^ ^34^ one on ERAS^35^ and four^36^ ^37^ ^38^ on other interventions (table 1). The latter included implementation of guidelines to improve obstetric care, increase vaginal delivery rate by increasing access to neuraxial analgesia, management guidelines, multifaceted educational activities and a package of QI activities at intervention sites (online supplemental appendix 2 provides a summary of each study).

### Analysis of implementation strategies

**Figure 2:**
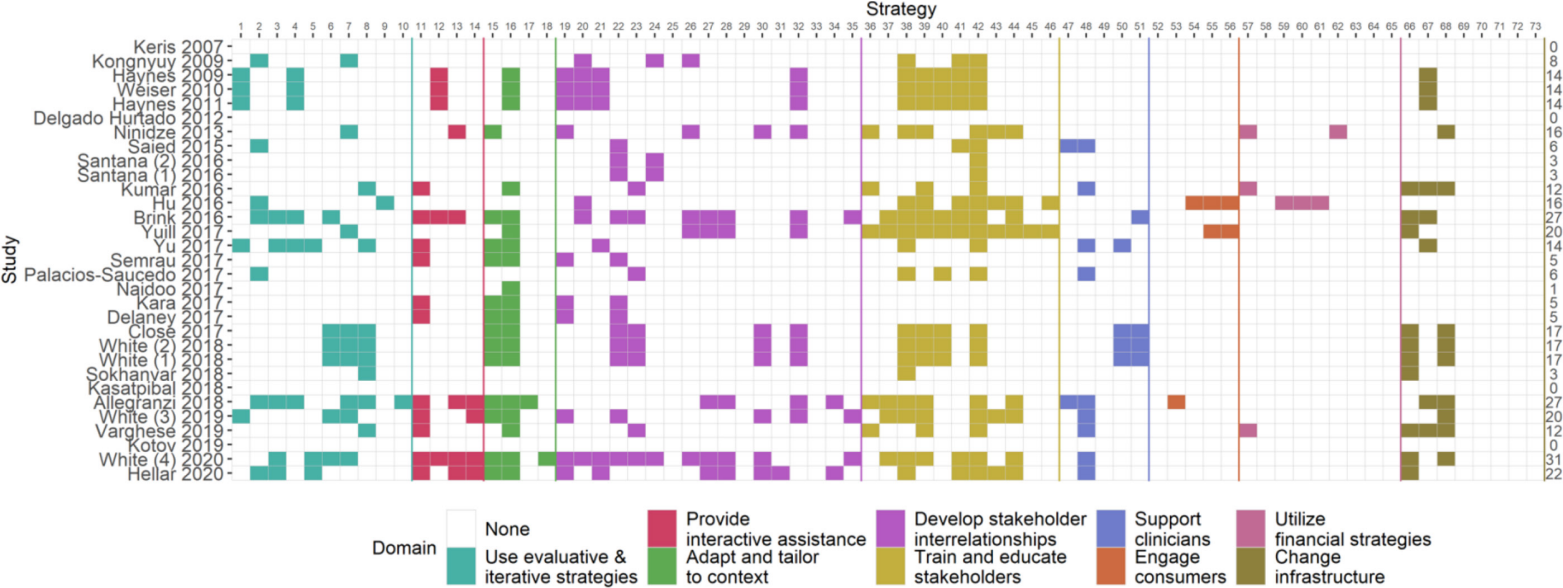
ERIC implementation strategies^19^ (shown by the nine domains) reported in each study. ERIC, Expert Recommendations for Implementing Change.

Figure 2 shows the ERIC implementation strategies used. Out of 73 different implementation strategies, 59 strategies were reported as being used across all 31 studies included in the final analysis. The median number of strategies used was 12 (range=0–31, IQR 4–17). Strategies that were most commonly reported across all included studies were from three out of nine ERIC domains: ‘train and educate stakeholders’, ‘develop stakeholder relationships’, and ‘use evaluative and iterative strategies’.

### Analysis of implementation outcomes

**Figure 3:**
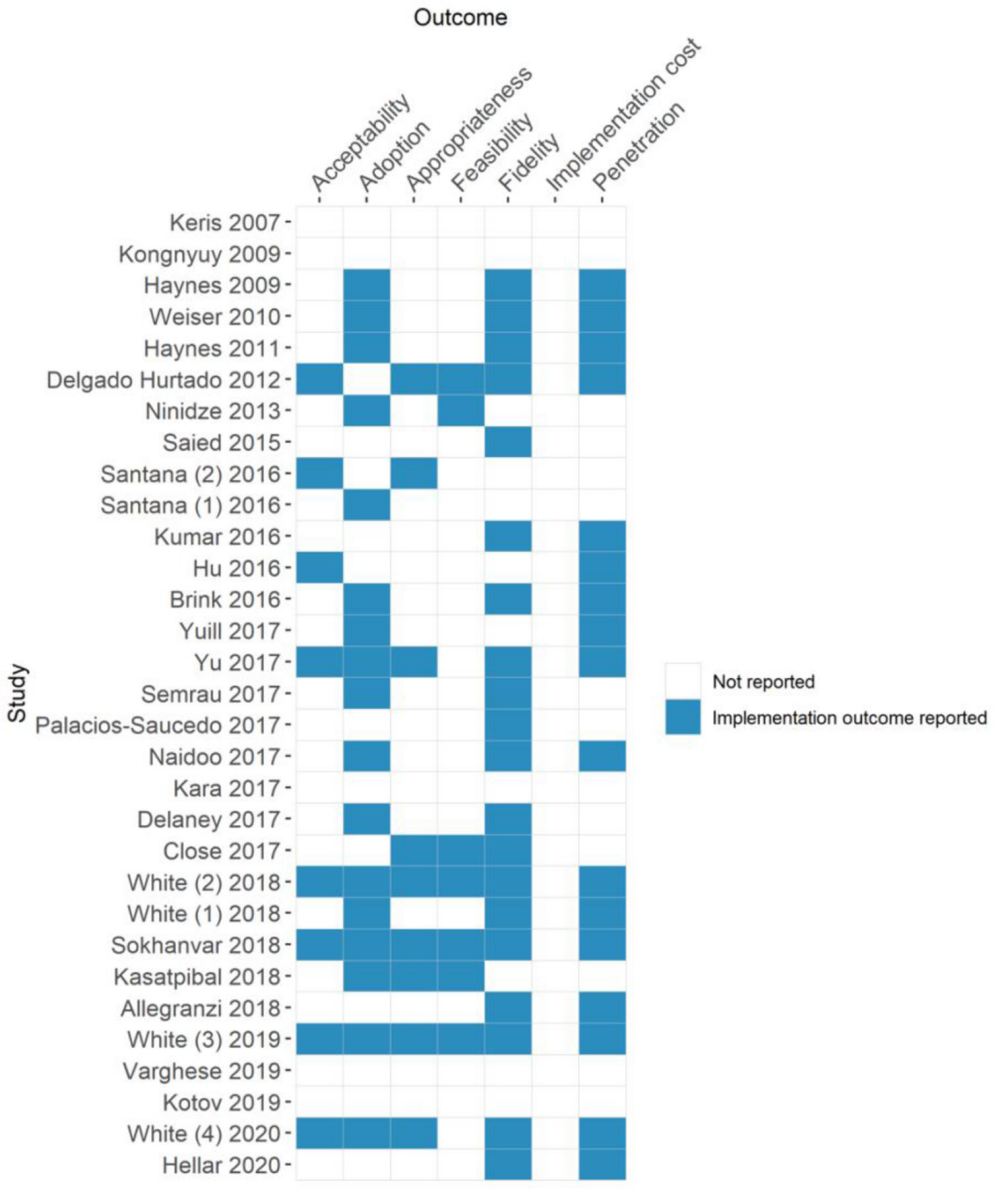
International implementation outcomes^20^ reported in each study.

Twenty-four out of 31 studies reported one or more implementation outcomes (figure 3). The most reported implementation outcomes were fidelity (64.5%, 20 of 31), adoption (54.8%, 17 of 31) and penetration (54.8%, 17 of 31). No study reported on implementation cost or sustainability. Median length of time between QI implementation and outcome evaluation was 6 months (range=1–108 months, IQR 5–18 months). As an implementation outcome, sustainability is not defined. We pragmatically defined it as 12 months.

### Analysis using scale-up frameworks

**Figure 4:**
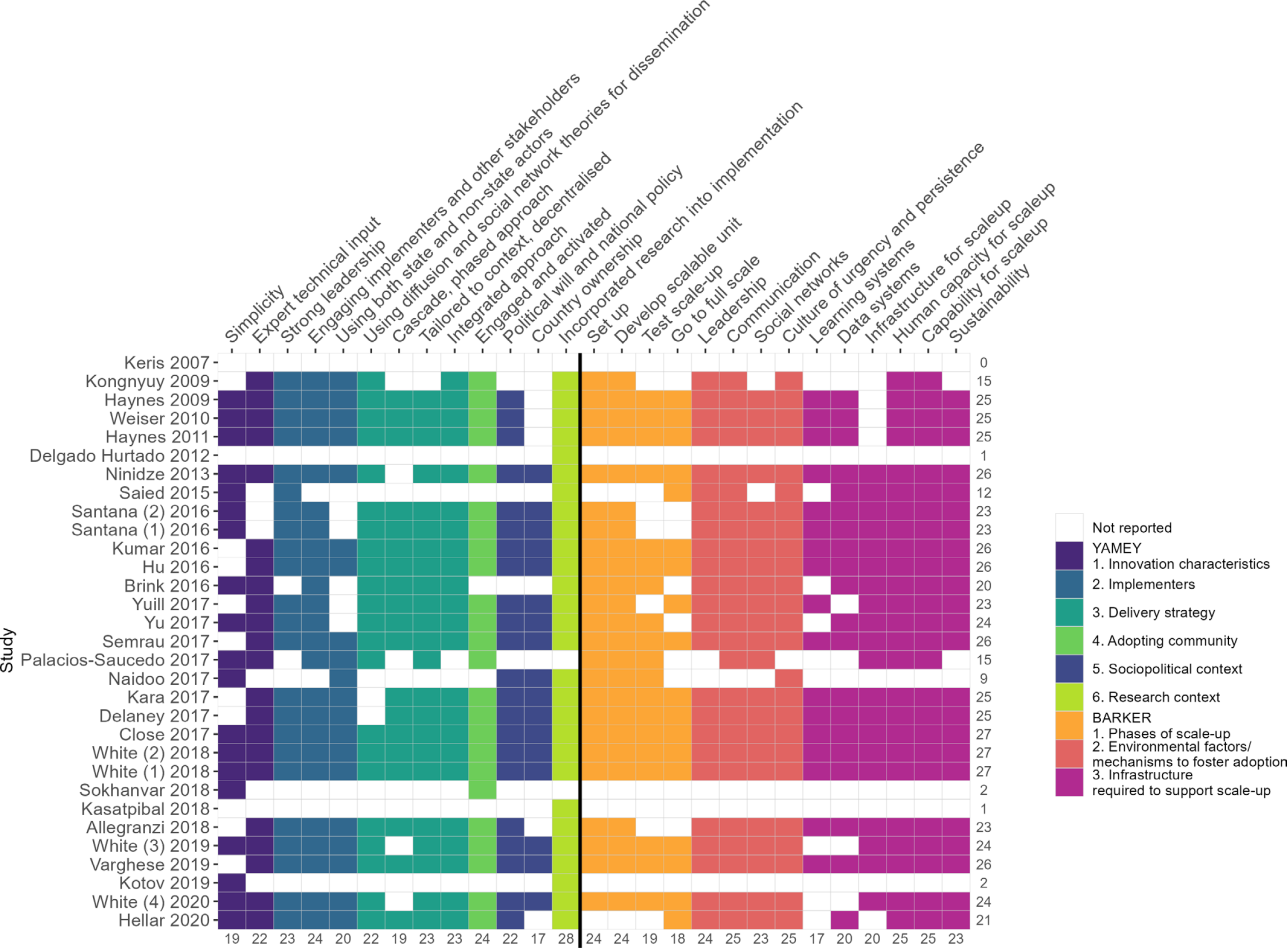
Components of Yamey^27^ and Barker *et al*^11^ scale-up frameworks reported.

The components of both scale-up frameworks (Yamey and Barker *et al*)^11 27^ were widely reported across all included studies (Yamey: median 10, range 0–12, IQR 7–11; Barker *et al*: median 13, range 0–15, IQR 9–15) (figure 4). Three^39–41^ out of 31 studies (9.6%) reported all of the discrete strategies from the Yamey (n=13) and Barker *et al* (n=14) frameworks. From the two frameworks combined, 22 (71%) of the studies reported more than 20 discrete strategies.^39–60^ However, no study made explicit reference to either the Yamey or the Barker *et al* frameworks.

## Discussion

To our knowledge, this is the first systematic attempt to assess the scale-up of perioperative point-of-care patient safety interventions in LMICs using established implementation frameworks. While a wide variety of QI interventions are reported as being implemented in LMICs, we found that very few (31 of 137) have been taken to scale in three or more sites. Of the interventions taken to scale, the WHO Surgical Safety Checklist is by far the most common (21 of 31) with only 5 of 31 broadly speaking related to AMS (including reduction of SSIs) and ERAS scale-up reported in a single study.

Over a decade ago in 2007, the WHO described the lack of scale-up of proven interventions to be a major failure of healthcare globally,^8^ and called for a focus on ways to increase the impact of proven interventions to benefit more people and foster policy development on a sustainable basis.^8^ Our results suggest there is much work still to be done. Innovative clinicians in surgery-designing technologies and process to improve surgical quality and safety cannot continue to operate with an implicit theory of spread—namely, that interventions proved to be successful in pilot studies or trials, will rapidly spread widely to positively impact population health simply through publication, market forces or communication networks.^5^ In LMICs, even when healthcare leaders and/or clinicians are aware of promising interventions, their ability to implement them is often severely constrained by limited time, resources and skill. There are several important steps between learning about a new concept, meaningful implementation in one’s own setting and subsequent widespread scale-up to other settings. Most innovative technologies and processes must be spread actively, not passively, or they may not be scaled effectively at all.^61^

The Yamey^27^ and Barker *et al*^11^ scale-up frameworks were designed to ensure active spread of innovations and they offer a guide to the key drivers for change, outlining the steps to take. We propose that they are intuitive to understand and apply for clinicians; and they may be considered as both process models and determinant frameworks for scale-up for implementation scientists. The individual components of both Yamey^27^ and Barker *et al*^11^ were widely reported across all studies in our review with little difference in between them making it impossible to recommend the use of one framework over the other. However, neither framework was referenced directly. This suggests that while the component parts of both scale-up frameworks may be intuitive, the frameworks themselves are less known. It could be argued that so long as scale-up occurs, it does not matter whether theoretical frameworks are cited. However, since the literature shows both high-profile failures of scale-up in the UK spanning more than a decade,^6 7 62 63^ as well as notable successes,^64 65^ and our results show scale-up is sparse in LMICs, we, along with others,^16^ propose that using theoretical models to guide the implementation process deserves more consideration. Theoretical models are based on observations and can highlight both barriers and facilitators to successful implementation and scale-up—but also, importantly, reasons for success or failure. We argue that mix of positive and negative results when evidenced interventions are taken to scale, in both HICs and LMICs, requires some theoretical thinking regarding, for instance, organisational and behavioural factors that may help (when present) or hinder (when absent) the success of a scale-up effort.

Theorising about what may work and reasons for it will help to develop the evidence base around the effectiveness and appropriateness of implementation and scale-up strategies in perioperative care interventions (and indeed more widely). We would further argue that, even if some scale-up models appear intuitive, they can still be useful by acting as a checklist for design, implementation and evaluation; help make the process more efficient; and reduce reliance on clinicians’ or organisations’ memory/skill set. Given that the failure of proven innovations to reach those who could benefit from them is not just a clinical failure but also a moral one as it compounds the inequity and injustice that already exists between HICs and LMICs. Theoretical models, we argue, are necessary for understanding the implementation process (including where such a process may fail) and, as a result, assisting in scaling up safety and quality measures. The standardisation that they bring allows comparison between studies and comprehensive analysis of the facilitators and barriers to scale up. If clinicians and academics are to contribute to population-wide health improvements, then it is time to pay as much attention to the mechanisms of scale-up as to the evaluation of an innovation’s effectiveness.

The median number of ERIC implementation strategies reported in our review was 12 (out of a total of 73 strategies identified in the evidence base^19^), and all studies reported at least one implementation strategy. This is higher than that reported by White *et al* in a recent systematic review specifically focused on the WHO Surgical Safety Checklist implementation in LMICs^29^: that study showed that most checklist implementations in LMICs were single site, the median number of strategies used per study was 4 and a quarter (12 of 47) of all WHO Checklist studies reported no implementation strategies. One explanation for the greater number of strategies we found could be that when interventions are taken to scale, a larger implementation effort is necessary; hence, more attention is paid to the implementation process, which in turn leads to better reporting of the implementation strategies used.

It remains unclear which of the 73 strategies listed in ERIC are most important for successful scale-up. Evidence from the WHO Checklist implementation (ie, not scale-up per se) suggests that strategies from the following five ERIC domains: ‘train and educate stakeholders’, ‘adapt and tailor to context’, ‘provide interactive assistance’, develop stakeholder relationships’ and ‘support clinicians’ are likely to be the most important for success. Our results suggest that in the context of scale-up, strategies from the three domains: ‘train and educate stakeholders’, ‘develop stakeholder relationships’, and ‘use evaluative and iterative strategies’ may be determinants of success. The importance of stakeholders is demonstrated by two domains common to both the WHO Checklist implementation and broader QI scale-up: the domains of ‘train and educate stakeholders’ and ‘develop stakeholder relationships’ together represent almost 40% of all ERIC strategies. This adds further weight to the suggestion that stakeholder influence is a key driver of implementation and especially scale-up. Our results further show that strategies in the domain ‘use evaluative and iterative strategies’ were used more commonly than the two domains found in White *et al*’s review^29^: ‘adapt and tailor to context’ and ‘provide interactive assistance’. This may be because when an intervention is applied initially at a single site, then the adaption to context and ongoing interactive assistance is important, (ie, adaptability, acceptability and adoption); once this has been tested in a single or pilot site setting, and the intervention then progresses to scale up, other factors such as those covered under the use of evaluative and iterative strategies become more prominent for robust and lasting scale-up (ie, high fidelity, sustainable scale-up). This is an interpretation, which requires empirical evaluation. Importantly, both the Yamey and Barker *et al* scale-up frameworks include incorporating research into the implementation and the use of learning systems and data systems, which are important in applying iterative strategies.

The concept of scalability is relatively loosely defined and can be confused with the ability to widen the reach of an intervention, and with scant attention to continued robust performance under routine conditions (ie, fidelity and sustainability).^66^ In our review, fidelity was reported in two-thirds of studies and the median length of time from implementation to evaluation of scale-up was 6 months.

### Limitations

This review has limitations. We did not exclude any study based on quality and therefore several potential biasing and confounding elements must be considered. Most of our data come from scale-up of the WHO Checklist, which means that the applicability of our findings to other interventions, such as ERAS and AMS, may be limited. Only two databases were searched to identify eligible studies and although we hand-searched the reference lists of included studies and review articles, it is possible that some studies were missed. There was likely under-reporting of the implementation strategies used and we did not contact individual study authors directly for further details. There may also be bias on the part of the data extraction teams in their interpretation of study methodology and mapping that to discrete implementation and scale-up strategies. Additionally, publication bias is probable and because there is no requirement to record implementation efforts a priori, the quality of the totality of implementation efforts globally may be less good than those published in per-reviewed journals and included in this study. The WHO Checklist, ERAS and interventions targeting SSI all have a strong evidence base in LMICs as well as HICs, but not all HIC interventions will be as transferable or as effective in low-resource settings so our results should be applied cautiously to interventions where evidence of clinical effectiveness in LMIC context is lacking. The World Bank Country and Lending Group classification is updated every fiscal year, and we used the classification from 2021 which means that countries that moved into the high-income classification over the last 60 years would have been excluded from the study. Lastly, our review (and any review on scale-up strategies for surgical interventions) rests on the assumption that scaling up benefits is ‘good’. This may be logical; however, scale-up may also lead to the scale-up of unintended harmful consequences; our study did not examine this.

This review also has several strengths. It describes and quantifies which perioperative QI interventions have been implemented in LMICs, specifically those taken to scale, and identifies explicitly the implementation methodologies used in scale-up. While most studies pertain to the WHO Checklist, future studies could focus on scale-up of other commonly applied interventions such as ERAS and interventions targeting AMS including the prevention of SSIs.

Despite a growing realisation in recent years of the role of implementation science in bridging the gap between research, policy and practice,^16 67 68^ our study suggests that use of theory-driven implementation approaches remains underused and is a missed opportunity in QI work globally. On a more positive note, since interventions such as ERAS and AMS (including reducing SSIs) are currently under-represented in LMICs, we feel we are faced with a golden opportunity for using theoretical implementation approaches to increase our understanding of the relationship between contextual factors and success and to help identify facilitators and barriers to scale up. Based on this review and recent reviews of WHO Checklist implementation^29^ and audit and feedback interventions to support antibiotic guidelines and reduce SSIs^28^ in LMICs, we propose two key recommendations. First, implementers should emphasise active stakeholder engagement when scaling perioperative QI interventions—including frontline clinicians, local academics and policymakers/managers. Second, we recommend broadening the generally clinician-driven QI implementation efforts to include a wider multidisciplinary team^69^ that includes implementation and improvement scientists (or at least a cadre of staff with expertise in change management, QI and behaviour or organisational change).

## Conclusion

If lives can be saved by improving the quality and safety of surgical care, then clinicians and academics need to urgently address the lack of widespread scale-up of proven QI interventions, especially in LMICs. The surgical safety community has a moral and ethical duty to ensure that any new practice of merit is actively shared and implemented with those in greatest need. Improving the understanding of the process and strategies of scale-up among clinicians, health system leaders and policymakers may be one place to start.

## Data Availability

Data are available upon reasonable request.
