# Supplementary material for: Scaling up of safety and quality improvement interventions in perioperative care: a systematic scoping review of implementation strategies and effectiveness
Source: BMJ Glob Health. 2022 Oct 26;7(10):e010649. doi: 10.1136/bmjgh-2022-010649 (PMC9615995; doi:10.1136/bmjgh-2022-010649)
Supplement: Supplementary data [file bmjgh-2022-010649supp001.pdf]

SUPPLEMENTARY FILE

Scaling up of safety and quality improvement interventions in perioperative care in low and middle income countries: a systematic scoping review of strategies and effectiveness of implementation.

LIST OF SUPPLEMENTARY MATERIALS

Appendix 1: Implementation science: basic concepts and definitions

Appendix 2: Summary of characteristics of studies meeting final (stage 2) inclusion criteria

Appendix 3: Search strategy for the Medline/EMBASE database:

Appendix 4: Eight gold-standard implementation outcomes as defined by the implementation science evidence-base[19] and adopted by the WHO[16]

Appendix 5: Definitions of implementation strategies, ERIC framework[17]

White MC, *et al. BMJ Global Health* 2023; 7:e010649. doi: 10.1136/bmjgh-2022-010649

Appendix 1: Implementation science: basic concepts and definitions

IMPLEMENTATION SCIENCE: BASIC CONCEPTS & DEFINITIONS

DEFINITIONS

Sometimes described as 'delivery science', implementation science focuses on the application of clinical and health research into care pathways. Implementation science focuses on the development of methods and techniques that can be applied to accelerate and enhance the uptake of evidenced practices into routine healthcare settings – including at the frontline of clinical care, within organisational settings and health policy-making. Implementation science supports innovative approaches to identifying, understanding, and overcoming barriers to the adoption, adaptation, integration, scale-up and sustainability of evidence-based interventions, tools, policies, and guidelines. Development and validation of evaluation methods and metrics that capture the speed and quality of intervention or programme implementation fall within the core remit of the science.

SCALE UP

Scale-up is often not precisely defined and sometimes the term appears to be used interchangeably with implementation science. here we adopt the definition of scale up proposed by the WHO, which defines the term as 'efforts to increase the impact of innovations successfully tested in pilot or experimental projects so as to benefit more people and to foster policy and programme development on a lasting basis'.

SUB HEADING

Historically, implementation science emerged in the aftermath of the evidence-based medicine movement; whereas conceptual development of scale-up approaches emerged as part of global health and global development. There is substantial overlap between scale-up and implementation science definitions and concepts- hence for the purposes of this study we use frameworks and taxonomies that span both fields.

IMPLEMENTATION STRATEGIES

These are defined as methods or techniques that can be applied to support the adoption, implementation, and sustainability of evidence-based interventions, programmes or policies. The most recent and widely used taxonomy of implementation strategies within healthcare is the ERIC taxonomy – which was derived based on systematic evidence review and an expert consensus process. The ERIC taxonomy identifies 73 different implementation strategies that can be sued within healthcare settings, which are grouped thematically into several categories.

IMPLEMENTATION OUTCOMES

The concept of 'implementation outcomes' was developed to supplement clinical (i.e. typically patient-level) and service outcomes and facilitate the design and evaluation of clinical studies with implementation components and also pure implementation studies.

Implementation outcomes have been defined as the effects of deliberate and purposive actions to implement new treatments, practices, and services. The most well-established taxonomy of the outcomes currently includes the following concepts: acceptability, feasibilty, appropriateness, adoption, fidelity, coverage, costs, and sustainability. It is important to note that these outcomes can (and arguably should) be collected to reflect both the clinical programme or intervention being implemented and also the implementation strategies that are used for the implementation. Choice of focus of the outcomes will depend on study/evaluation priorities.

Definitions of each implementation strategy are shown in Supplementary material page 8. Definitions of each implementation outcome in Supplementary material page 9.

White MC, *et al. BMJ Global Health* 2023; 7:e010649. doi: 10.1136/bmjgh-2022-010649

Appendix 2: Summary of characteristics of studies meeting final (stage 2) inclusion criteria

S. No.	First author surname	Year of publication	Description of intervention	Country and (study sites =n)	Study Design	Key outcomes
1	Allegranzi[31]	2018	Evaluation of a before-after cohort study on the effect of a multimodal intervention on SSIs in Africa	Kenya, Uganda, Zambia (n=5)	Observational - prospective	SSI cumulative incidence significantly decreased post intervention, from 8.0% (95% CI 6.8-9.5; n=129) to 3.8% (3.0-4.8; n=70; p<0.0001), and this decrease persisted in the sustainability period (3.9%, 2.8-5.4; n=35).<br>A substantial improvement in compliance with prevention measures was consistently observed in the follow-up and sustainability periods.
2	Brink[32]	2016	Multimodal bundle- learning sessions for pharmacists and pharmacy managers on Netcare PAP guidelines, core measures for improvement, formation for multidisciplinary teams to conduct regular QI cycles	South Africa (34 Hospitals)	Pre and post implementation study	Sustained decrease in the SSI rate of 19.7% to a mean rate of 1.97 (95% CI 1.79–2.15) (P=0.0029)

3	Close[33]	2017	Checklist implementation programme: (P1) 3-day training course in each hospital; (P2)informal telephone follow-up 6 weeks later to members of hospital staff identified during the training as important to sustainable implementation; and (P3) an in-person follow-up visit at 3–4 months post course to evaluate participant experiences and organisational change.	Madagascar (21 Hospitals)	Mixed methods	Unclear from abstract
4	Delaney[34]	2017	Comparison of adherence to 18 practices of the Better Birth Trial (which is studying the effectiveness of a Safe Childbirth Checklist-cantered intervention on maternal and neonatal harm) recorded by both coaches and independent observers. 8-month staggered coaching intervention over a 20 month period in 60 public health facilities.	India (120 hospitals)	Process evaluations embedded in an RCT	birth attendants' behaviour during 5,971 deliveries was observed where 35 of 39 essential birth practices had achieved>90% adherence in the presence of a coach, compared with only 7 of 39 practices during the first month. Without a coach present, birth attendants 'average adherence to practices and checklist use was 24 percentage points lower than when a coach was present (range:1% to 62%).

5	Delgado Hurtado[35]	2012	Anonymous self-responding questionnaire administered to investigate the knowledge, acceptance, current use, teamwork and safety climate appreciation one year after the implementation of the checklist.	Guatemala (3)	Observational retrospective: structured questionnaire	Between 73.7% and 100% of nurses in public and private hospitals, respectively, reported the checklist had been used either always or almost always in the general elective surgeries they had participated in during the current year. Despite high acceptance of the checklist among personnel, gaps in knowledge about when the checklist should be used still exist.
6	Haynes[36]	2009	2 step checklist implementation program. (P1) baseline data collection (P2) checklist training + implementation (P3) data collection	Canada, India, Jordan, New Zealand,	Observational - prospective	Implementation of the checklist was associated with concomitant reductions in the rates of death and complications among

			post checklist implementation	Philippines, Tanzania, United Kingdom, USA (sites = 8)		patients at least 16 years of age who were undergoing noncardiac surgery in a diverse group of hospitals. The rate of death was 1.5% before the checklist was introduced and declined to 0.8% afterward (P=0.003).
7	Hellar[37]	2020	team-based approach employed in a low-resource setting in Tanzania, reviewed reported data from facility registers supplemented by direct observation data by mentors to evaluate the use of the WHO SSC over a period of one year	Tanzania (n=40)	Observational - prospective	utilisation of checklist improved 98%, the proportion of correctly filled checklists has increased
8	Hu[38]	2016	Increase access to labour neuraxial analgesia in China. Driven by Chinese expats now working in the US through volunteer led training weeks. Covered 31 hospitals in 7 years.	China (n=31)	Mixed methods	24/31 hospitals had 24/7 anaesthetic coverage. Labour epidural rates are >50%, c-section rates have reduced, transfusion rates have reduced and neonatal outcomes have improved.
9	Kara[39]	2017	Surgical checklist (incl. maternal/birth)	India (n=120)	Qualitative study (describes the	Implementation of Better Birth strategy of WHO SCC with coaching can be a method for achieving change in facility based

					program)	childbirth care.
10	Kasatpibal[40]	2018	Surgical checklist (incl. maternal/birth)	Thailand (n=33)	Prospective surveillance	increase in near miss detection and reduction in wrong sites and wrong patients. Crude analysis showed that surgeon-specific feedback was not associated with a change in SSI [relative risk (RR) . 1.01, 95%CI . 0.77e 1.33].
11	Keris[41]	2007	Retrospective Cohort Study - national guidance produced 2001; evaluation of practice and outcomes before and after	Latvia (n=32)	Observational-retrospective	Implementation of the Guidelines was associated with a statistically significant decrease of hospital case fatality rate in TBI patients. reduction of HCFR from 3.7% during 1998-2000 to 2.6% during 2002-2004 (relative risk 0.72; 95% confidence interval 0.67-0.76; p = 0.03)
12	Kongnyuy[42]	2009	Developed protocol/Recommendations for the identification and management of obstructed labour in Malawi. Covered 3 districts and 8	Malawi (n= 8)	Observational-retrospective	Criterion based audit can improve the management of obstructed labour in countries with limited resources. draining of

			hospitals.			urinary bladder (70.5 vs. 90.2%; P = 0.022), administration of broad spectrum antibiotics (72.7 vs. 90.2%; P = 0.039), commencement of Caesarean section within 1 hour or delivery of the foetus within 2 h of diagnosis (38.6 vs. 61.0%; P = 0.023), and maintaining an observation chart (45.5 vs. 61.0%; P < 0.001).
13	Kotov[43]	2019	Multicentre prospective observational study of Enhanced Recovery After Surgery Protocol utilised in patients undergoing radical cystectomy - 4 centres	Russia (n=4)	Observational-prospective	Despite the use of the ERAS protocol, radical cystectomy has a high frequency of complications (up to 70%); most of them are Clavien I-II. A 30-days mortality rate is 5.2%, and re-hospitalization is required in 9.7% cases.
14	Kumar[44]	2016	Implementation of a modified WHO safety in childbirth checklist in 101 hospitals in India in association with the ministry of health. Outcomes were compared with 99 facilities who didn't implement the checklist. Smaller	India (n=8)	Observational-retrospective	The SCC was used by providers in 86 % of 240 deliveries observed in the eight intervention facilities. Providers in the intervention group significantly adhered to practices included in the SCC than providers

			scale study whereby 8 facilities had a simple implementation package to see whether this improved adherence.			in the comparison group controlling for baseline scores and confounders.
15	Naidoo[45]	2017	Pre-intervention surgical outcomes were assessed. Training of healthcare personnel then the MSSCL was implemented. Post-intervention surgical outcomes were assessed.	South Africa (n=18)	RCT	Significant improvements in the adverse incident rate per 1 000 procedures, postoperative sepsis, referral to higher levels of care and unscheduled return to the operating theatre in cases with checklist. Greater reductions in maternal mortality in hospitals that were good implementers of the MSSCL
16	Ninidze[46]	2013	Programme of education (and protocol development) for patients and staff regarding the safe use of regional anaesthesia for obstetrics in 5 Georgian hospitals over a 3 year period. Including looking at supply chain logistics.	Georgia (n=5)	Mixed methods	A structured program of education and quality improvement led to an increase in the use of regional anaesthesia for vaginal and caesarean deliveries. Achievements were sustained during periods of economic and political turmoil.
17	Palacios-Saucedo[47]	2017	Implemented a programme aimed at modifying the prescribing behaviour in	Mexico (n=6)	Observational-prospective	303 surgical patients, 218 prophylactic antibiotics regimens. Inappropriate treatment

			surgical prophylaxis across 6 hospitals in a region of Mexico, involving printed, audio-visual and electronic materials, and assessed the impact on the use of antibiotics			commencement was reduced to 84.1% (180/214) (P<0.001), inappropriate regimens to 75.3% (162/215) (P=0.03), inappropriate dosages to 51.2% (110/215) (P<0.001), and use of restricted antibiotics to 8.3% (18/215) (P=0.003).,
18	Saied[48]	2015	Implementation of an antimicrobial stewardship programme in 5 tertiary hospitals.	Egypt (n=5)	Observational-prospective	The optimal timing of the first dose improved significantly in 3 hospitals, increasing from 6.7% to 38.7% (P <.01), from 2.6% to 15.2% (P <.01), and from 0% to 11% (P <.01). All hospitals showed a significant rise in the optimal duration of surgical prophylaxis, with an overall increase of 3%-28% (P < .01)
19	Santana (1)[49]	2016	Linked to Santana 2016 (2). This presents the results of a safety attitudes questionnaire completed via interview in the Pre and Post	Brazil (n=3)	A prospective cross-sectional study	Regarding checklist adherence in Period II, ‘‘Patient identification’’ significantly improved in the stage ‘‘Before induction of

			intervention periods. 472 healthcare professionals were surveyed (Pre-post intervention response rate 82% vs 75%); 257 before the intervention and 215 post intervention.			anaesthesia''. ''Allergy verification'', ''Airway obstruction verification'', and ''Risk of blood loss assessment'' had low adherence in all three hospitals.
20	Santana (2)[50]	2016	Linked to Santana 2016 (2). This presents the results of a safety attitudes questionnaire completed via interview in the Pre and Post intervention periods. 472 healthcare professionals were surveyed (Pre-post intervention response rate 82% vs 75%); 257 before the intervention and 215 post intervention.	Brazil (n=3)	Observational-prospective	Despite the variability in checklist item compliance in the surveyed hospitals, WHO checklist implementation as an intervention tool showed good adherence to the majority of the items on the list. Nevertheless, motivation to use the instrument by the surgical team with the intent of improving surgical patient safety continues to be crucial
21	Semrau[51]	2017	Matched-pair, cluster-randomized, controlled trial in 60 pairs of facilities across 24 districts of Uttar Pradesh, India, testing the effect of the Better Birth program (8-month coaching-based implementation of the Safe Childbirth	India (n=120)	RCT	Significant clinical management changes: Hysterectomy within 7 days 19/79,705 (<0.1) vs 18/77,252 (<0.1); RR (95% CI) 1.00 (0.45–2.13); p = 0.95. Blood transfusion within 7 days 640/79,697 (0.8)

			Checklist) on a composite outcome of perinatal death, maternal death, or maternal severe complications within 7 days after delivery.			vs 625/77,254 (0.8); RR (95% CI) 0.99 (0.69–1.43); p= 0.97.
22	Sokhanvar[52]	2018	8 Hospitals in Iran. Conducted an awareness, attitudes and acceptance questionnaire following what appeared to be a nationally driven implementation project placing responsibility on individual hospitals (not by the same authors).	Iran (n=8)	Qualitative	Despite the variability in checklist item compliance in the surveyed hospitals, WHO checklist implementation as an intervention tool showed good adherence to the majority of the items on the list. Nevertheless, motivation to use the instrument by the surgical team with the intent of improving surgical patient safety continues to be crucial
23	Varghese[53]	2019	As above (Kumar 2016), but implementation strategy was expanded across 100 facilities, and this study looks at the mortality effect for stillbirths an early neonatal deaths at the 19 intervention centres that had special new-born care units.	India (n=7)	Observational - retrospective	Reduction in stillbirths by 11.39%, and reduction in early neonatal deaths by 10.35%. Overall reduction in mortality by 11.16%.

24	White (1)[54]	2018	3 day dynamic educational course on WHO checklist implementation in Madagascar. Checklist implemented; at 6 weeks follow-up call to trouble shoot problems. Success of implementation evaluated at 3-4 months	Madagascar (n=21)	Mixed-methods	reach went from almost zero to 78%, participant years in practice, hospital size or surgical volume did not predict checklist use. Checklist use was associated with counting instruments, but not other key safety steps.
25	White (2)[55]	2018	Follow-up study to White (1) 2018. 14/21 original hospitals in Madagascar visited over a 4 week period by 4 person evaluation team. Primary outcome = SSC use measured by a self-report questionnaire. Secondary outcomes = use of 6 steps; team behaviour utilising WHOBARS; association between checklist utilisation; impact od sustained SSC; and barriers to sustained SSC utilisation. Data collected in 3 ways - self report questionnaire, WHOBARS and focus groups	Madagascar (n=14)	Mixed-methods	74% of participants reported sustained checklist use 12-18 months following nationwide implementation, with associated improvements in job satisfaction, culture and compliance with safety procedures.
26	White (3)[56]	2019	3 day MDT training in WHO checklist in Benin and four month follow up. Subsequent	Benin (n=36)	Mixed-methods	reach increased from 31% to 88% at 3-4 months and was sustained at 86% after 12-

			evaluation of checklist use at 12–18 months with questionnaires and focus groups.			18months. High fidelity use (median WHOBARS 5/7), use of basic safety process ranged from 85-99%. Improvement in hospital safety culture, and high scores for acceptability, adoption, appropriateness and feasibility. Intervention used 31/36 CFIR constructs
27	White (4)[57]	2020	Implementation of WHO SSC: 1) problem id - lack of routine checklist use; 2) multifaceted implementation of WHO SSC following strategy including - 3-day multidisciplinary training course, coaching, facilitated leadership engagement, and support networks); 3) outcome evaluation 4 months postintervention	Cameroon (n=25)	Mixed-methods	reach increased from 20-56%, high fidelity 79-95%, and 4.5/7 using WHOBARS,
28	Yu[58]	2017	China	China (n=4)	Mixed methods	The revised SSC content was simplified from 34 to 22 items. Anaesthetists achieved widespread recommendation as SSC coordinators. Completion rates of all stages

						reached over 80· 0 per cent at all sites (compared with 10· 2–59· 5 per cent at the sign-out stage in the baseline survey).
29	Yuill[59]	2017	Multinational team of physicians invited to Armenia to observe and establish standards of obstetric anaesthetic care over a period of 9 years. The aim was to develop national protocols and guidelines and achieve a minimum standard of care throughout Armenia. There was focus on neuraxial anaesthesia for caesarean section 9and for labour analgesia but only in city centre hospitals due to availability of anaesthetists in rural hospitals).	Armenia (n=21)	Mixed Methods	Increased use of neuroaxial anaesthesia for Caesarian delivery, increased use of epidural labour analgesia, and national obstetric anaesthesia practice guidelines have been established
30	Haynes (2)[60]	2011	Before and after study of surgical safety checklist implementation program. (P1) baseline data collection (P2) checklist training	Canada, India, Jordan, New Zealand,	Observational-prospective	Clinicians in the preintervention phase (n=281) had a mean SAQ score of 3.91 / 5 while the postintervention group (n=257)

			+ implementation (P3) data collection post checklist implementation. Aim was to assess the relationship between changes in clinician attitude (using the Safety Attitude Questionnaire, SAQ) and changes in postoperative outcomes	Philippines, Tanzania, United Kingdom, USA (n=8)		had a mean of 4.01 /5 (p=0.0127). The degree of improvement in mean SAQ score at each site correlated with a reduction in postoperative complication rate (r=0.7143, p=0.0381). 80% of respondents considered the checklist easy to use.
31	Weiser[61]	2010	Before and after study of the impact of implementing the surgical safety checklist implementation program for urgent surgical procedures. Collected data for 1750 consecutively patients undergoing urgent noncardiac surgery in 8 diverse hospitals around the world; 842 underwent urgent surgery-defined as an operation required within 24 hours of assessment to be beneficial-before introduction of the checklist and 908 after introduction of the checklist.	Canada, India, Jordan, New Zealand, Philippines, Tanzania, United Kingdom, USA (n=8)	Observational-prospective	The complication rate was 18.4% (n=151) at baseline and 11.7% (n=102) after the checklist was introduced (P=0.0001). Death rates dropped from 3.7% to 1.4% following checklist introduction (P=0.0067). Adherence to 6 measured safety steps improved from 18.6% to 50.7% (P<0.0001)

White MC, *et al. BMJ Global Health* 2023; 7:e010649. doi: 10.1136/bmjgh-2022-010649

Appendix 3: The search strategy for the Medline/EMBASE database:

1. surg* or Operating theatre or Operating rooms or Intraoperative procedures or intraoperative period or Preoperative period or preoperative procedures or Perioperative period or perioperative procedure or Postoperative period or postoperative procedure or periop*
2. anes* or anaes* or exp anaesthesia
3. 1 or 2
4. checklist or triage or early warning score or exp protocol or exp guidelines or practice guidelines or quality improvement or patient safety or pathway or bundle or fasting, thromboprophylaxis, pt admission, airway, and failure to rescue
5. (Afghan* or Albania* or Algeria* or Samoa* or Angola* or Antigua* or Barbuda* or Aruba or Arubian* or Argentin* or Armenia* or Azerbaijan* or Bahrain* or Bangladesh* or Belarus* or Beliz* or Benin* or Bhutan* or Bolivia* or Bosnia* or Herzegovin* or Botswana* or Brazil* or Bulgaria* or Burkina Faso or Burundi* or Cambodia* or Cameroon* or Cabo Verd* or Cape Verd* or Central African Republic or Chad* or Chile* or China or Chinese or Colombia* or Comoros or Comorian or Congo* or Cote d'Ivoire or Ivory Coast or Costa Rica* or Croatia* or Cuba or Cuban or Cyprus or Cypriot* or Czech* or Dominica* or Djibouti* or Ecuador* or Egypt* or El Salvador* or Eritrea* or Estonia* or Ethiopia* or Fiji or Gabon* or Gambia* or Gaza* or Georgia* or Ghana* or Gibraltar* or Greece or Greek or Grenad* or Guam or Chamorro* or Chamoru or Guatemala* or Guinea* or Guyana* or Haiti* or Hondura* or Hungar* or India* or Indonesia* or Iran* or Iraq* or Isle of Man or Mann or Manx or Jamaica* or Jordan* or Kazakh* or Kenya* or Kiribati* or Korea* or Kosovo* or Kyrgyz* or Lao* or Latvia* or Leban* or Lesotho* or Liberia* or Libya* or Liechtenstein or Lithuania* or Macao or Macau or Macanese or Macedonia* or Madagasca* or Malawi* or Malay* or Maldiv* or Mali or Marshall Island* or Maurit* or Mexic* or Micronesia* or Moldova* or Mongolia* or Montenegr* or Morocc* or Mozambi* or Myanm* or Burm* Namibia* or Nepal* or New Caledonia* or Nicaragua* or Niger* or Pakistan* or Palau* or Panam* or Paraguay* or Peru* or Philippin* or Filipin* or Poland or Polish or Portug* or Puerto Ric* or Romania* or Russia* or Rwand* or Samoa* or Sao Tome* or Principe or Saudi Arab* or Senegal* or Serbia* or Seychell* or Sierra Leone* or Slovak* or Slovenia* or Solomon* or Somalia* or South Africa* or Sri Lanka* or Kitts

or Nevis or Lucia* or Vincent or Grenadines or Sudan* or Surinam* or Swazi* or Syria* or Tajikistan* or Tanzania* or Thai* or Timor* or Togo* or Tonga* or Trinidad* or Tobag* or Tunisia* or Turkey or Turkish or Turkmen* or Tuvalu* or Uganda* or Ukrain* or Uruguay* or Uzbekistan* Vanuatu* or Venezuela* or Vietnam* or Yemen* or Zambia* or Zimbabwe* or Sub-Sahara* or Sahara* or Africa* or SSA or Asia* or Pacific or South America* or Latin America* or Central America* or East Europe* or Eastern Europe* or LIC or LICs or LAMIC or LAMICs or LMIC or LMICs or MIC or MICs or UMIC or UMICs).ab,ti.

6. ("scaling up" or "scaled up" or "scale-up" or "up-scaling" or "upscaling").ti,ab. OR (scalability or scalable or "at scale").ti,ab
   OR (spread adj5 (innovation* OR intervention* OR technolog* OR practice OR care)).ti,ab. OR ((bring* or brought or taking or take* or increas* or going or implement*) adj5 scale)).ti,ab.
7. Final equation: Surg*/Anes* AND Peri-op QI interventions AND LMICs AND Scale-up (3 AND 4 AND 5 AND 6)

Appendix 4: Eight gold-standard implementation outcomes as defined by the implementation science evidence-base [19] and adopted by the WHO [16]

**Implementation Outcome**	**Definition**
Acceptability	The perception among stakeholders that the intervention is acceptable
Appropriateness	The perceived fit, relevance, or compatibility of the intervention for a given practice setting, provider, or consumer; and/or perceived fit of the intervention to address a particular issue or problem
Feasibility	The extent to which an intervention can be successfully used
Adoption	The intention, initial decision, or action to implement an intervention
Fidelity:	Extent to which an intervention gets applied as originally designed / intended
Implementation costs:	Costs of the delivery strategy, including the costs of the intervention itself
Penetration:	Extend to which eligible patients/population actually receive intervention

White MC, *et al. BMJ Global Health* 2023; 7:e010649. doi: 10.1136/bmjgh-2022-010649

Appendix 5: Definitions of implementation strategies, ERIC framework[17]

Implementation strategies are 'methods and techniques used to enhance the adoption, implementation and sustainability of a clinical programme, practice or intervention'[19]

Implementation strategy domain	Discrete strategies within the domains
I. Use Evaluative and Iterative Strategies (n=10)	Assess for readiness and identify barriers and facilitators (1), Audit and provide feedback (2), Conduct cyclical small tests of change (3), Conduct local needs assessment (4), Develop a formal implementation blueprint (5), Develop and implement tools for quality monitoring (6), Develop and organize quality monitoring systems (7), Purposely re-examine the implementation (8), Stage implementation scale-up (9), Obtain and use patients/consumers and family feedback (10)
II. Provide Interactive Assistance (n=4)	Provide local technical assistance (11), Facilitation (12), provide clinical supervision (13), Centralize technical assistance (14)
III. Adapt and Tailor to Context (n=4)	Use data experts (15), use data warehousing techniques (16), Promote adaptability (17), Tailor strategies (18)
IV. Develop Stakeholder Interrelationships (n=17)	Develop an implementation glossary (19), Model and simulate change (20), Capture and share local knowledge (21), Conduct local consensus discussions (22), Build a coalition (23), Develop academic partnerships (24), Identify early adopters (25), Inform local opinion leaders (26), Involve executive boards (27), Obtain formal commitments (28), Promote network weaving (29), Use advisory boards and workgroups (30), Use an implementation advisor (31), Visit other sites (32), Identify and prepare champions (33), Recruit, designate and train for leadership (34), Organize clinician implementation team meetings (35)
V. Train and	Provide ongoing consultation (36), make training dynamic (37), conduct

	Educate Stakeholders (n=11)	educational meetings (38), conduct educational outreach visits (39), conduct ongoing training (40), create a learning collaborative (41), develop educational materials (42), distribute educational materials (43), Shadow other experts (44), Work with educational institutions (45), Use train-the-trainer strategies (46)
VI.	Support Clinicians (n=5)	Develop resource sharing agreements (47), remind clinicians (48), revise professional roles (49), facilitate relay of clinical data to providers (50), Create new clinical teams (51)
VII.	Engage Patients and Service Users (n=5)	Increase demand (52), intervene with patients/consumers to enhance uptake and adherence (53), involve patients/consumers and family members (54), prepare patients/consumers to be active participants (55), Use mass media (56)
VIII.	Utilize Financial Strategies (n=9)	Access new funding (57), alter incentive/allowance structures (58), alter patient/consumer fees (59), develop disincentives (60), Fund and contract for the clinical innovation (61), make billing easier (62), Place innovation on fee for service lists/formularies (63), use capitated payments (64), Use other payment schemes (65)
IX.	Change Infrastructure (n=8)	Change accreditation or membership requirements (66), Mandate change (67), start a dissemination organization (68), change service sites (69), change liability laws (70), change physical structure and equipment (71), change record systems (72), Create or change credentialing and/or licensure standards (73).
